# Villous adenoma of the renal pelvis: a common entity at an uncommon location

**DOI:** 10.4322/acr.2021.283

**Published:** 2021-05-06

**Authors:** Sushma Bharti, Vikarn Vishwajeet, Himanshu Pandey, Poonam Abhay Elhence

**Affiliations:** 1 All India Institute of Medical Sciences (AIIMS) Jodhpur, Department of Pathology & Lab Medicine, Jodhpur, Rajasthan, India; 2 All India Institute of Medical Sciences (AIIMS) Jodhpur, Department of Urology, Jodhpur, Rajasthan, India

**Keywords:** Kidney pelvis, adenoma, villous, hydronephrosis, pyonephrosis

## Abstract

Villous adenoma is uncommonly seen in the urogenital tract and is even more rarely seen in the upper urinary tract and renal pelvis. Like colorectal adenomas, these neoplasms can transform into adenocarcinoma. The preoperative diagnosis is challenging due to their frequent association with hydronephrosis. Herein, we present the case of a villous adenoma of the renal pelvis in a 62-year-old man presenting with recurrent urinary tract infection. The computed tomography scan showed marked hydronephrosis but no suspicious mass in the right kidney. A laparoscopic right nephrectomy was performed. Gross examination revealed a dilated renal pelvis with an irregular exophytic lesion in the renal pelvis’s upper surface. The histopathological examination showed slender, elongated villi with thin fibrovascular cores, consistent with villous adenoma morphology. Isolated villous adenomas have a favorable prognosis. However, the pathologist should undertake a search for an invasive component.

## INTRODUCTION

Villous adenoma is usually located in the gastrointestinal tract and may be a risk lesion for malignant transformation. The occurrence of villous adenoma outside the gastrointestinal tract lacks germline mutation. Several studies and case reports showed the occurrence of villous adenoma in the urinary bladder.^1^^,^^2^ However, the occurrence of villous adenoma in the urogenital system outside the urinary bladder is exceptionally uncommon, and only a few cases of villous adenoma are described involving the renal pelvis.^3^^,^^4^ Herein, we report a case of villous adenoma of the renal pelvis in an elderly patient.

## CASE REPORT

A 62-year-old male sought the emergency department complaining of right flank pain and high-grade fever with chills and rigor over the last three days. He had an increased frequency of micturition with dysuria. His medical history included similar symptoms over the past three years with pyonephrosis, right kidney failure, and diabetes mellitus on medication. On examination, the patient was febrile; however, he was conscious and cooperative. Pulse rate was increased. Blood pressure was normal. No pallor, icterus, cyanosis, lymphadenopathy, or edema was seen. The cardiovascular and respiratory system was normal except for increased heart rate and increased respiratory rate. Abdominal examination revealed tenderness in the right flank region.

The routine investigation revealed a deranged renal function test with a serum creatinine of 2.55mg/dl (reference range [RR]; 0.6-1.2mg/dl) and serum urea of 55mg/dl (RR, 15-40mg/dl). The hemogram revealed mild anemia with normal leukocytes and platelet counts. Urine microscopy showed plenty of polymorph leukocytes. Ultrasonography revealed moderate to marked hydronephrosis in the right kidney. The renal cortex width varied between 6mm and 9mm. The pelvicalyceal system was dilated and showed a thick and particulate fluid corresponding to approximately 300-400ml. The left kidney showed a small and simple cortical cyst in the lower pole. The cortical echogenicity was mildly altered. The urinary bladder wall was irregular and mildly thickened with punctate echoes in the lumen. Renal DMSA (dimercaptosuccinic acid) scan with 5mCi 99m-Technetium revealed enlarged hydronephrotic right kidney with moderately impaired cortical function and evidence of cortical scarring. The differential function of the right kidney was 25%. The left kidney was normal in size with preserved cortical function. The diagnosis of pyonephrosis of the right kidney was considered with the clinical and radiological findings.

A percutaneous nephrostomy was considered and attempted. However, it was not successful due to the drainage tube’s repeated blockage by a thick mucus-like material. In the absence of significant improvement, a laparoscopic right nephrectomy was performed. Intraoperatively, a dense perinephric adhesion was found with diffuse edema in the surrounding tissue planes. The right kidney was mobilized from the abdominal wall and lower pole. After the hilum and the upper pole dissection, the kidney was removed through a small lower abdominal incision. Antibiotic regimen with ceftriaxone was started on the day of surgery. The patient was discharged on the 5^th^ post-operative day.

**Figure 1:**
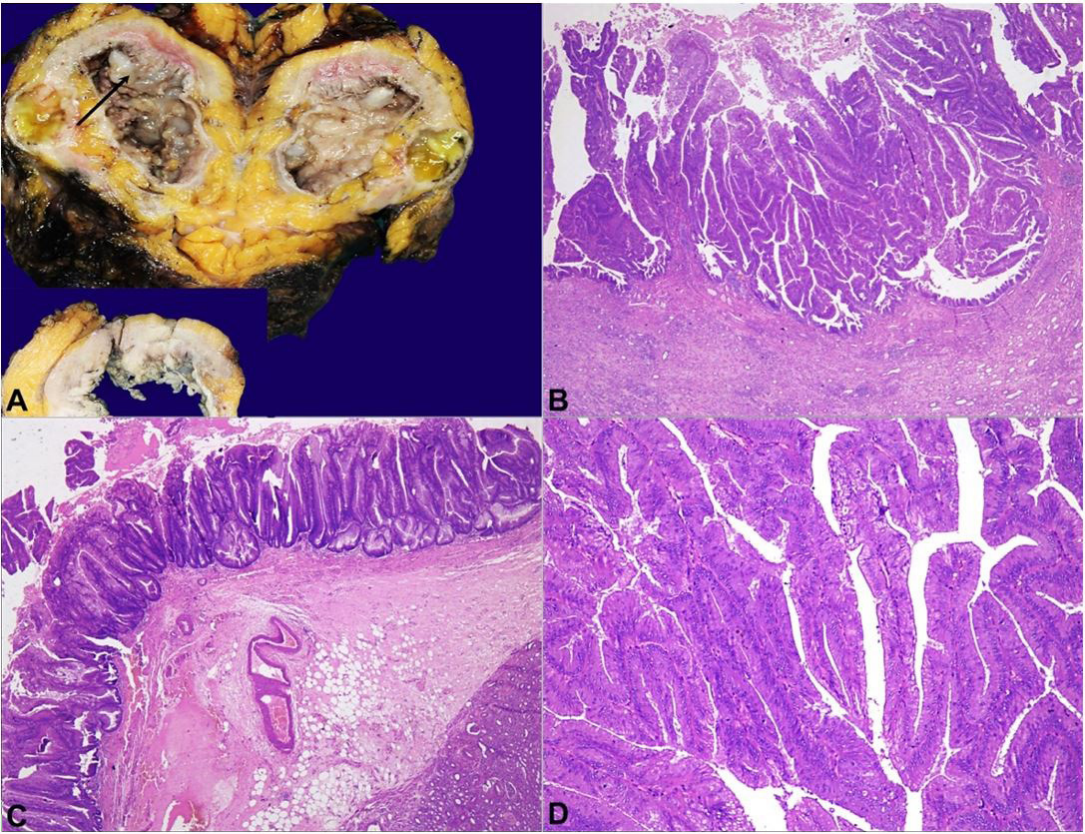
**A** – Gross photograph of the right kidney showing markedly dilated pelvis with solid white exophytic growth in the upper pole (arrow). Mucus was seen over the growth. Inset shows a close-up view of the lesion; **B** – Photomicrograph showing adenomatous polyp composed of closely packed slender villi (hematoxylin and eosin [H&E], X40); **C** – These villi are seen replacing the urothelium. No invasion in stroma seen (H&E, ×40); **D** – Closely packed slender villi lined by columnar epithelium with low-grade dysplasia (H&E, ×100).

**Figure 2:**
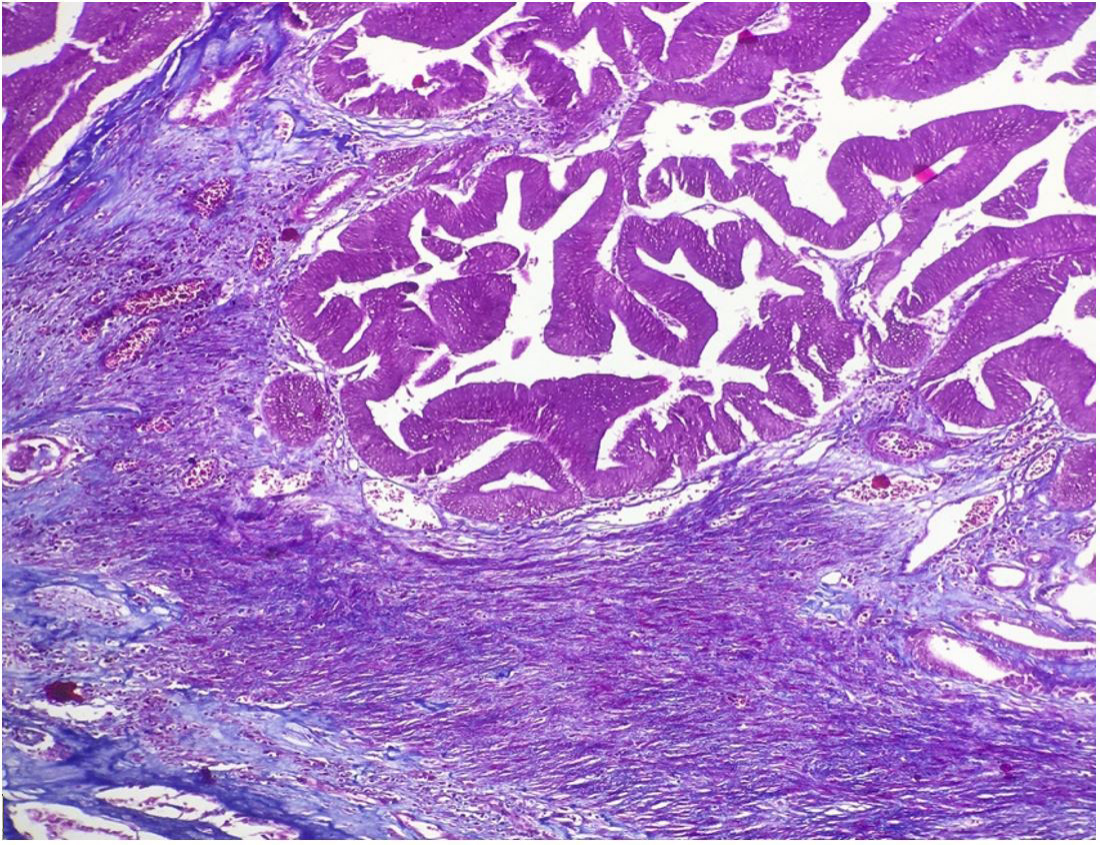
Photomicrograph shows a sharp demarcation of the tumor from the stroma, and the absence of muscle layer involvement indicates a lack of invasion. (Masson trichrome, 40X).

Grossly, the kidney was enlarged and measured 14x9x4.5cm. The ureter and pelvicalyceal system were markedly dilated and filled up with pus. The renal parenchyma was thinned out. Near the upper pelvis, an irregular papillary growth filling the major calyx was identified. The lesion was covered with mucus and measured 3.5x2x2cm (Figure 1A). The lesion’s microscopic examination showed a polypoid tumor composed of slender and elongated villi with a thin fibrovascular core. These villi showed irregular folding and outpouchings and were lined by epithelial cells with nuclear stratification, hyperchromasia, and frequent luminal mitoses, indicating dysplastic changes. Goblet cells were also noted. No invasion in the underlying stroma was noted (Figure 11D, and Figure 2).

**Figure 3:**
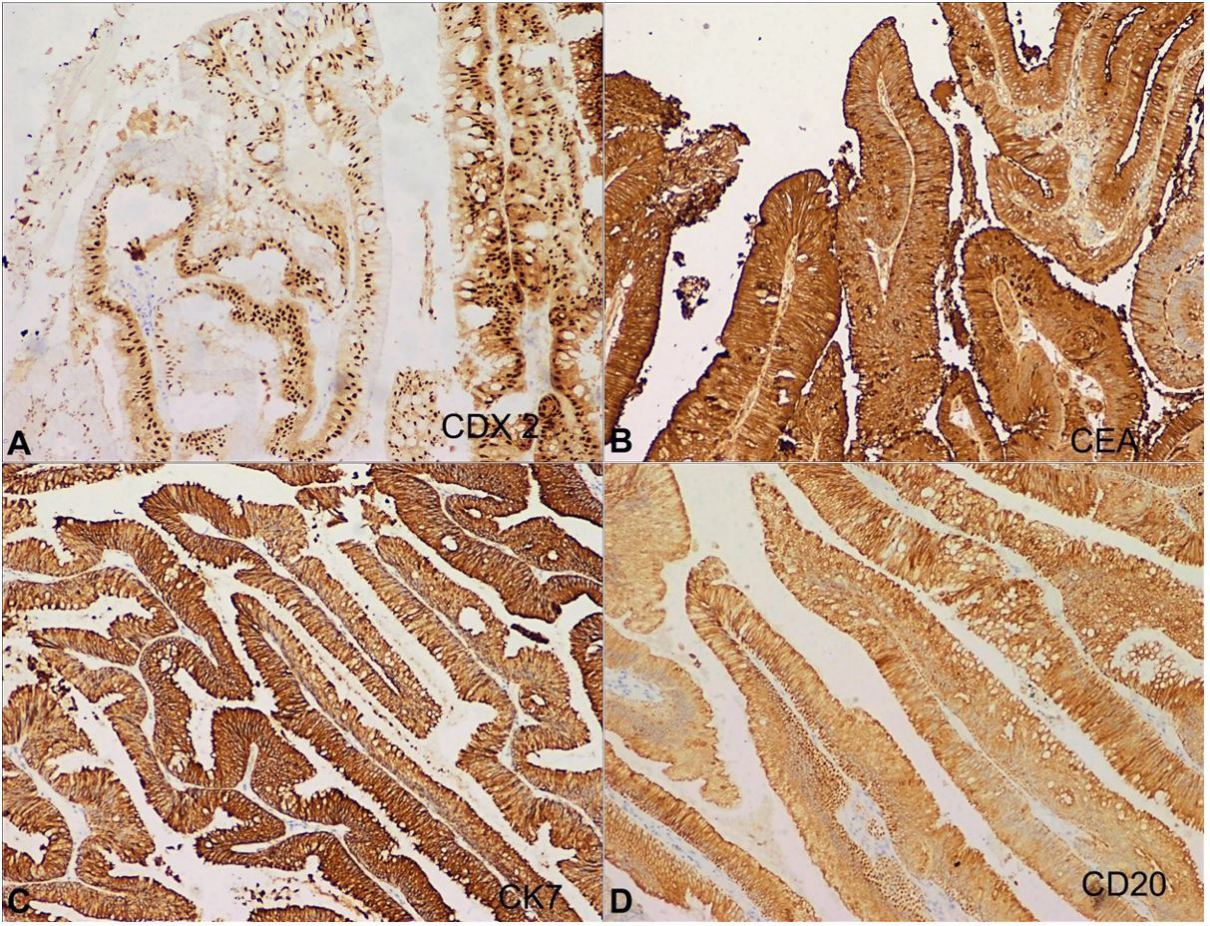
Immunohistochemistry. Tumor cells showing strong nuclear reactivity for CDX2 (**A**), membranous positivity for CEA (**B**), cytoplasmic positivity for CK7 (**C**) and CK20 (**D**) [A-D, X100].

On immunohistochemistry, the tumor cells showed strong cytoplasmic positivity for CK7 and CK20, nuclear positivity for CDX2, and membranous positivity for CEA (Figure 3). These cells were negative for p63. The remaining renal parenchyma showed multiple abscesses involving both the medullary tissue as well as the cortex. A diagnosis of pyonephrosis with villous adenoma of the renal pelvis was rendered. The patient was doing well after 6 months of follow-up with no fresh complaints.

## DISCUSSION

Villous adenomas are commonly seen in the gastrointestinal tract, i.e., in the colon and rectum, followed by the stomach, small intestine, papilla of Vater, gallbladder, and appendix. However, they can also be seen in the urinary tract. In 1978, Assor^5^ described the first urothelial villous adenoma in the urinary bladder, and Park et al.^6^ reported the first case of renal pelvis villous adenoma in 2002, who used the term “muconephrosis”. Villous adenomas of the renal pelvis are very uncommon, and till now, only 14 cases have been reported in the English literature.^3^^,^^4^^,^^6^^-^^16^ (Table 1). They are mostly seen in the middle age to elderly male patients. The patients present as asymptomatic hematuria and lower irritative voiding symptoms. Fever was eventually present, along with lower back or lumbar pain, abdominal discomforts and weight loss, and intermittent mucosuria. Involvement of the right kidney is more frequently seen. Of the 14 cases reported in the literature, the left kidney involvement was noted in only one-fifth of the cases.

**Table 1:** Summary of villous adenoma of renal pelvis reported in literature till now

Author	Age/ gender	Presentation	NLT	HDN	Sd	Size	Follow up
Stoykov et al.^7^	61/M	Pain and discomfort in right lumbar region, intermittent mucosuria	Y	Y	Rt.	NA	NA
Nayak et al.^8^	52/M	Incidentally found during transplant work-up	N	N	B/l	3.3cm	On treatment at 3 months
Llu et al.^9^	70/M	Percutaneous fistula with jelly-like yellow mucus	Y	Y	Rt.	NA	NED at 6 months
Bote et al.^10^	60/M	Dull aching pain in right lumbar region	-	Y	Rt.	3X2cm	NED at 10 months
Dong et al.^11^	61/M	Hematuria	Y	Y	Rt.	4cm	NED at 3-4 years
	65/M	Right upper quadrant mass	-	Y	Rt.	rough, velvet-like and gray-colored inner wall of pelvis	
Huang et al.^3^	54/M	Dysuria, flank pain, mucosuria	Y	Y	Rt	Multiple, 2cm largest	NED at 12 months
Patel et al.^12^	45/F	Low grade fever and right flank pain	-	Y	Rt.	NA	NED at 3 months
Sagnotta et al.^13^	81/F	Right hypochondriac mass and recurrent fever	-	Y	Rt.	Entire pelvis mucosa with papillary excrescences	NA
Hudson et al.^14^	81/F	Difficulty voiding and mucosuria	-	Y	Rt.	8.5cm & 1cm	NED at 1and ½ months
Karnjanawanichkul et al.^15^	73/M	abdominal discomfort and a palpable abdominal mass, mucosuria	Y	Y	Rt.	Patch of sessile mass at pelvis	NA
Bhat and Chandran^4^	52/M	Left abdominal pain and mass	Y	Y	Lt.	0.5cm	NED at 1 yr
Fudge et al.^16^	NA/M	Back pain and weight loss	Y	N	Rt.	Multiple, largest 2.5cm	NA
Park et al.^6^	79/M	Fever and right flank pain	-	Y	Rt	3.5cm	NA

B/l: Bilateral; ESRD: End Stage Renal Disease HDN: Hydronephrosis; H/o: History of, Lt: Left; N: no; NA Not available: NED: No evidence of disease; NLT= Nephrolithiasis; Rt: Right, Sd= side, Y: yes.

Villous adenomas were invariably associated with hydronephrotic kidneys, which was attributed to obstructive uropathy caused by stones in the urinary tract in most cases, detected either at presentation or had the previous history of nephro/urolithotomy. In one case, there was a history of an augmentation colo-cystoplasty.^8^ The proposed mechanism implicated is the metaplastic changes of the urothelium lining in response to chronic irritation and inflammation. These may be caused by recurrent urinary tract infections or calculi.^2^

The diagnosis of villous adenoma in the upper urinary tract is challenging because of the lack of specific clinical or radiological features. These lesions in the upper urinary tract, per se, are uncommon and are often difficult to recognize. Another feature that makes the diagnosis difficult is their frequent association with hydronephrotic kidney secondary to calculi or recurrent urinary tract infections. In all cases reported in the literature, a preoperative diagnosis of villous adenoma of the renal pelvis was not considered on imaging studies. In these studies, nephrectomy was done because of hydronephrosis and/or nephrolithiasis.^4^^,^^9^^,^^14^^,^^15^ In the present case, the villous adenoma was not clinically suspected as no defined mass lesion was identified in the renal pelvis on radiological testing. Of note, these lesions are known to produce a copious amount of mucous. The presence of mucosuria provides an essential clue for suspecting the diagnosis of villous adenoma.

However, mucosuria is an uncommon presentation and was noted in only 20% of the cases.^3^^,^^4^^,^^6^^-^^16^ In the present case, repeated blockage of the drainage tube inserted following percutaneous nephrostomy was due to thick mucus passage. However, mucus production may be seen in rare xanthogranulomatous pyelonephritis cases, pseudomyxoma peritonei, and direct invasion of gastrointestinal tract malignancies.^6^^,^^17^ Further, in cases presenting with mucosuria, the specimen collected can be subjected to cytology. The cell block can be prepared from the sample and can be tested for immunocytochemistry, and can aid in the pre-operative diagnosis. However, mucosuria is not observed in every case, and hence a definite diagnosis relies on pathological examination of the nephrectomy specimen. Grossly, almost all cases had enlarged kidney size with the markedly dilated renal pelvis. The tumor was detected either as a single localized mass-like lesion or as a patch of velvety or papillary excrescences replacing pelvicalyceal lining.^3^^,^^4^^,^^6^^-^^16^

Microscopic examination reveals features similar to the primary villous adenoma of the gastrointestinal tract with thin, slender villi lined by basally located nuclei and abundant pale eosinophilic mucin-filled cytoplasm. Variable degree of admixed goblet cells is also noted. A mild to moderate degree of atypia may be noted in the epithelial cells. On immunohistochemistry, the tumor cells show positivity for CEA, CK20, CDX-2, and CK7. CK7 positivity is variable and is reported in 56% of the urinary tract villous adenomas.^2^ In the present case, IHC results are concordant with these previous reports. The expression of IHC markers similar to those expressed by primary gastrointestinal villous adenoma reflects the urinary tract’s metaplastic potential into intestinal phenotype.

The prognosis of villous adenoma with adequate surgical treatment is excellent. However, villous adenomas may harbor an invasive adenocarcinoma.^8^^,^^13^ One should exclude the invasive component after examining adequate sections. Special stains such as Masson trichrome and even immunohistochemical testing by desmin or smooth muscle actin may exclude the muscle layer involvement. Cheng et al.^2^ studied 23 cases of villous adenoma, 8 of them with coexistent adenocarcinoma. The study included villous adenoma of the urinary bladder. Patients with isolated villous adenoma in this study has neither any recurrence nor transformation into invasive adenocarcinoma during a follow-up of 9.9 years. Seibel et al.^1^ studied 18 cases of villous adenoma involving the urinary bladder, urachus, and prostatic urethra. In their study, pure villous adenoma was seen in 33% of the cases, while the remaining cases had either in –situ or invasive components of adenocarcinoma or urothelial carcinoma. Of the reported renal pelvis villous adenoma, 40% of the cases harbored invasive adenocarcinoma.^3^^,^^4^^,^^6^^-^^16^ Inadequate and improper management can lead to severe consequences on the prognosis of the patients. Hence, the pathologist must examine the specimen diligently to exclude invasive components.

## References

[1] Seibel JL, Prasad S, Weiss RE, Bancila E, Epstein JI (2002). Villous adenoma of the urinary tract: a lesion frequently associated with malignancy. Hum Pathol.

[2] Cheng L, Montironi R, Bostwick DG (1999). Villous adenoma of the urinary tract: a report of 23 cases, including 8 with coexistent adenocarcinoma. Am J Surg Pathol.

[3] Huang TY, Yang SF, Huang SP, Yeh HC, Li CC (2014). Villous adenoma of the renal pelvis: A case report and literature review. Urol Sci.

[4] Bhat S, Chandran V (2010). Villous adenoma of the renal pelvis and ureter. Indian J Urol.

[5] Assor D (1978). A villous tumor of the bladder. J Urol.

[6] Park S, Meng MV, Greenberg MS, Deng DY, Stoller ML (2002). Muconephrosis. Urology.

[7] Stoykov B, Kolev N, Dunev V, Genov P, Atanasov J, Mateva S (2020). A rare case of huge villous adenoma of the renal pelvis deforming the abdominal wall. Urol Case Rep.

[8] Nayak A, Depasquale B, Vergara N, Guzzo TA, Lal P (2019). Villous Adenoma Arising in the Native Bladder Mucosa and the Upper Urinary Tract With Coexisting Neuroendocrine Carcinoma Following Augmentation Cystoplasty. Int J Surg Pathol.

[9] Liu D, Tan J, Huang K, Jiang Z, He L, Yin G (2017). Villous adenoma in renal pelvis with manifestation of percutaneous fistula and mucus secretion. Urology.

[10] Bote SM, Siddiqui MA, Gite VA, Patil SR, Menon S (2016). Villous adenoma of renal pelvis with muconephrosis: a case report. Arch Int Surg..

[11] Dong C, Yang Y, Wu S, Chen G (2015). Clinicopathological analysis of two cases with pelvis villous adenoma and review of relevant literature. J Cancer Res Ther.

[12] Patel RD, Vanikar AV, Modi PR (2014). Mucinous cystadenocarcinoma of renal pelvis presenting as pyonephrosis. Saudi J Kidney Dis Transpl.

[13] Sagnotta A, Dente M, Socciarelli F, Cacchi C, Stoppacciaro A, Balducci G (2014). Primary adenocarcinoma of the renal pelvis: histologic features of a stepwise process from intestinal hyperplasia to dysplasia in a patient with chronic renal abscess. Int J Surg Pathol.

[14] Hudson J, Arnason T, Merrimen JL, Lawen J (2013). Intestinal type villous adenoma of the renal pelvis. Can Urol Assoc J.

[15] Karnjanawanichkul W, Tanthanuch M, Mitarnun W, Pripatnanont C (2013). Renal pelvic villous adenoma presented with mucusuria: report of a case and literature review. Int J Urol.

[16] Fudge KG, Glicklich D, Golestaneh L, Pullman J (2006). Villous adenoma in the native kidney of a renal transplant recipient. Transplantation.

[17] Shah VB, Amonkar GP, Deshpande JR, Bhalekar H (2008). Mucinous adenocarcinoma of the renal pelvis with pseudomyxoma peritonei. Indian J Pathol Microbiol.

